# Computational optical biopsy

**DOI:** 10.1186/1475-925X-4-36

**Published:** 2005-06-14

**Authors:** Yi Li, Ming Jiang, Ge Wang

**Affiliations:** 1 Department of Mathematics, University of Iowa, Iowa City, IA 52242, USA; 2 Bioluminescence Tomography Laboratory, Departments of Radiology and Biomedical Engineering, University of Iowa, Iowa City, IA 52242, USA

**Keywords:** Molecular imaging, optical imaging, fluorescent imaging, bioluminescent imaging, inverse problem, diffusion approximation

## Abstract

Optical molecular imaging is based on fluorescence or bioluminescence, and hindered by photon scattering in the tissue, especially in patient studies. Here we propose a computational optical biopsy (COB) approach to localize and quantify a light source deep inside a subject. In contrast to existing optical biopsy techniques, our scheme is to collect optical signals directly from a region of interest along one or multiple biopsy paths in a subject, and then compute features of an underlying light source distribution. In this paper, we formulate this inverse problem in the framework of diffusion approximation, demonstrate the solution uniqueness properties in two representative configurations, and obtain analytic solutions for reconstruction of both optical properties and source parameters.

## Introduction

Gene therapy is a breakthrough in the modern medicine, which promises to cure diseases by modifying gene expression. A key for development of gene therapy is to monitor the *in vivo *gene transfer and its efficacy in the mouse model. Traditional biopsy methods are invasive, insensitive, inaccurate, inefficient, and limited in the extent. To map the distribution of the administered gene, reporter genes such as those producing luciferase are being used to generate light signals within a living mouse, which can be externally measured [1]. A cooled highly sensitive CCD camera has been built to take a 2D view of expression of the bioluminescent reporter luciferases. Such a 2D image of photon emission is then superimposed onto a 2D visible light picture of the mouse for localization of the reporter gene activity. In addition to gene therapy, this new imaging tool has great potentials in various other biomedical applications as well. An *in vivo *bioluminescence tomography system integrated with an X-ray CT/micro-CT scanner is recently reported in [2,3]. The novel concept is to collect emitted photons from multiple 3D directions with respect to a living mouse marked by bioluminescent reporter luciferases, and reconstruct an internal bioluminescent source distribution based on *both *the outgoing bioluminescent signals and the CT/micro-CT volume of the mouse. Then, the 3D bioluminescent source distribution and the corresponding CT/micro-CT volume are registered of anatomical and pathological structures, such as the lung and various tumors.

Optical imaging of small animals based on fluorescent/bioluminescent probes promises great opportunities for translational research and eventually clinical applications, because fluorescent/ bioluminescent signals directly reveal molecular and cellular activities, and are sensitive, specific, non-ionizing, non-invasive and cost-effective. Pure optical imaging cannot detect the molecular activities triggered by biomarkers because the light generated are generally out of the visible spectrum. Despite the progress in optical molecular imaging of small animals, little research has been done for optical molecular imaging of patients. A light source induced by either fluorescence or bioluminescence probes is usually weak, and would be often deep inside a body should it be used in patients. Optical methods for in vivo imaging are all faced with the problem of limited transmission of light through tissues [[1], p. 237]. Because the human body absorbs and scatters photons in the visible and near infrared ranges with the mean-free-path in the sub-millimeter domain, such a source cannot be effectively detected on the body surface [4].

In this paper, we propose a computational optical biopsy (COB) method [5] to supplement and enhance the capabilities of fluorescent molecular tomography and bioluminescence tomographic, especially for their potential uses in patients. In order to detect the light source in a region of interest deep inside a subject, we can use a fiber-optical probe to detect the light source directly in the subject along one or multiple biopsy paths and next to compute the parameters or features of the embedded light source.

Several optical biopsy needle systems are already in operation [6-8], which have indicated the physical feasibility of this COB project. While similar to existing optical biopsy procedures in using fiber-optic probes [6-8], the proposed COB system and methods depend on not only optical devices but also advanced modeling and computation techniques to reconstruct an underlying source intensity distribution and extract its features of interest such as source center and effective intensity. There are several distinctions that substantiate our innovations. Our COB approach relies on sophisticated signal modeling and estimation from data collected along a number of biopsy paths, while other biopsy/endoscopic techniques perform direct anatomical imaging on speci c spots only. Our COB targets source intensity distributions triggered by probes instead of tissue/vascular properties that are concerned by other biopsy/endoscopic methods. Our COB intends to sense both fluorescent and bioluminescent sources, not just fluorescent sources as some optical endoscopic/spectroscopic techniques are designed for.

Mathematically we will Consider



where *u*_0 _is the average photon flux in all directions, , *μ*_*α *_are positive constants with *μ*_*a *_and *μ*_*s *_being the absorption and scattering constants respectively in  and *f *is either a measure or a *L*^∞ ^∩ *L*^1 ^function, which represents the light source.

## Single point source

**Figure 1:**
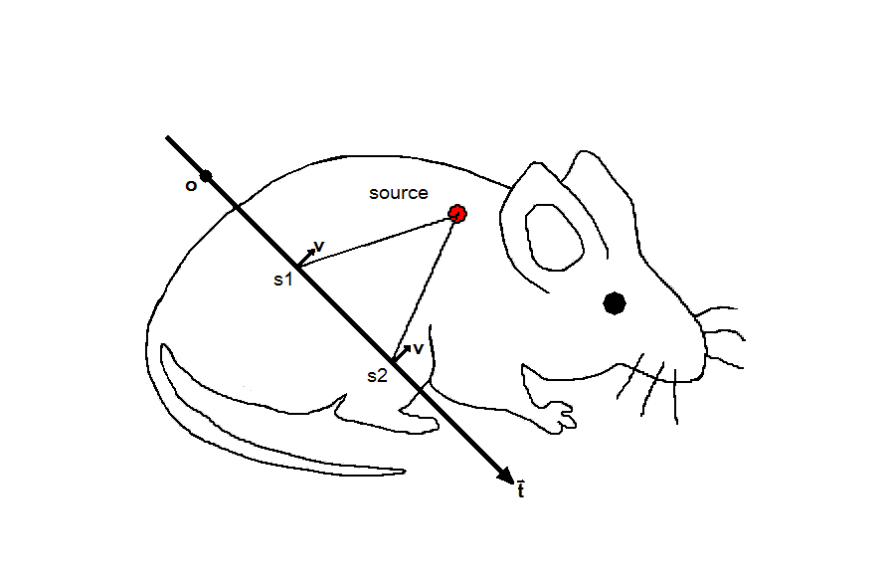
A needle insertion path, where *t* is the direction of the insertion, 0 one point along the insertion, *v* a direction in which the needle tip detects light, s_1_; s_2_ are points along the path

We will first consider the case where the source term , where *δ *is the Dirac operator. In this case we have



where *C *is a constant such that



or



i.e.,



Assume that the needle insertion follows a straight line *l *: , where  is the direction of the insertion and  one point along the insertion (Figure 1).

Let *υ *be a direction that needle tip detects light from. Then the measurement along the line *l *is given by



and  = (*t*_1_, *t*_2_, *t*_3_). *υ *= (*υ*_1_, *υ*_2_, *υ*_3_) which has a fixed angle *θ *with the needle direction , i.e., cos (*θ*) = *t*_1_*υ*_1 _+ *t*_2_*υ*_2 _+ *t*_3_*υ*_3_

such that



Due to the notation and translation invariant properties of (1) and the design of the insertion needle, we may assume, without loss of generality, that



so that *υ*_1 _= 0, (*υ*_2_*υ*_3_) = (cos *α*, sin *α*) and . Then (7) becomes



Now let .

Assuming that each insertion line can detect sources from two different angles *α*_1_, *α*_2 _such that *α*_1 _- *α*_2 _≠ *kπ*, *k *∈ ± , then



is non-singular. Therefore appropriate combination *α*_1 _and *α*_2 _depending on *γ *of the measurements *m*(*s*) would single out , so that without loss of generality, we may assume that



which are the measurements such that <*x *- *x*^*i*^, *υ *> = , *k *= 2, 3 after combinations of *α*_1 _and *α*_2 _mentioned above and define



### Theorem 2.1

(*Single point source*) *If N *= 1, *i.e.: if *, *then one insertion would uniquely determine λ, x^1^, and μ_*eff*_, provided that the insertion line does not go through the point of the source. (See remark.)*

### Proof

In this case since





Differentiate (13) with respect to *s*, we have





such that the critical point of  (*s*) uniquely identifies *x*_1_. Next with *x*_1 _identified, we obtain



or



And by taking the derivatives of  and evaluating them at *x*_1_, we obtain that





where *z *= *μ*_eff_*w*.

From (18) we obtain



and plugging it into (19) we have



If *f *(*z*) is monotone in *z *> 0, then (21) would give us a unique solution *z *> 0.

We compute and obtain



i.e., we have uniquely solved by the information from  the values of *μ*_eff_, *w *and therefore *λ *by (17).

Next if we let *x*_2 _= *w *cos *β*, *x*_3 _= *w *sin *β*, then we get



which could uniquely determine *β *by the sign conditions of , *k *= 2.3.

That is, by now, we are able to determine, (*x*_1_, *x*_2_, *x*_3_), *μ*_eff _and *λ*; i.e. complete information about the source.

### Remark

In case that the insertion line goes through the point of the source, which is verified by having each

{some single point}

then only the two components of the point source, orthogonal to the insertion can be identified, which is the best possible. On the other hand, in practice, such events would have probability zero!

## Single ball source

Next we consider ball sources, i.e., we assume that



where *χ*Ω is the characteristic function of Ω and .

For such source we have where



where

 – the total intensity of the source in the form of *λχ*_*B*(*o*, *r*)_.     (24)

Again we will discuss the single ball source first, i.e. *N *= 1, so that we have



### Theorem 3.1

(*Single ball source*) *If N *= 1 *; i.e. if u*_0 _*is given by *(*25*), *then one insertion would uniquely determine M *(*λ*_3_, *r*^1^), *x*^1 ^*and μ*_*eff*_; *in case the line stays outside of B *(*x*^1^, *r*^1^)*. In case the insertion line enters the interior of B *(*x*^1^, *r*^1^), then *x*^1^, *r*^1^, *λ*_1 _*and μ*_*eff *_*are uniquely determined by one insertion.*

### Proof

#### Case 1

If the insertion line (refer to Theorem 1.1 in Section 2) stays outside *B *(*x*^1^, *r*^1^), then the detected *u*_0 _is given by



i.e., *u*_0 _(*x*) behaves exactly as a single point source with an intensity of *M *(*λ*_1_, *r*^1^) and hence Theorem 2.1 guarantees the result.

#### Case 2

If the insertion line enters the support of the ball source *B *(*x*^1^, *r*^1^) but misses the center point *x*^1^, then again due to the rotation and translation invariant properties of (1), we may assume, without loss of generality that the insertion is given by (8) and *x*^1 ^= (*x*_1_, *x*_2_, *x*_3_) and hence the detection given by (6) is now

*m *(*s*)     (27)



where .

Again, assuming that each insertion line can detect sources from two different angles *α*_1_, *α*_2 _such that *α*_1 _- *α*_2 _≠ *kπ*, *k *∈ ± , then



is non-singular. Therefore appropriate combination of the two measurements depending only on *α*_1 _and *α*_2 _would single out  and , so that without loss of generality we may assume that

*m*_*k *_(*s*) = *φ *(*s*), where



If we let *x*_2 _= *w *cos *β*, *w*_3 _= *w *sin *β*, then



which would uniquely determine *β *by the above question and the sign conditions if , *k *= 2.3.

Define

 (*s*) = *m*_2 _(*s*)^2 ^+ *m*_3 _(*s*)^2 ^= *w*^2^*φ*^2 ^(*s*).     (30)

Note that since in this case we assume that the insertion line enters the interior of *B *(*x*^1^, *r*^1^) but misses the center *x*^1^, we have 0 <*w *<*r*^1 ^and the line would enter and leave *B *(*x*^1^, *r*^1^) when



which is exactly where our measurement *M*_*k *_(*s*), or equivalently *φ *(*s*) fails to be differentiable. Therefore let *s*_in _and *s*_out _be two points where we observe the jump discontinuity of ^1 ^(*s*).

Then we have



(32) together with (29) uniquely identify *x*_1 _and establish a relation between *r*_1 _and *w*. Then we are able to evaluate  (*x*_1_) to get



i.e.,



Next by taking the derivatives of  and evaluating them at *x*_1_, we obtain that





>From (35) we obtain



and plugging it into (36) we get



which can be veri ed to be an increasing function. Therefore (38) has a unique solution *z *and (37) then uniquely de nes the *w *which in turn defines *λ*_1 _uniquely by (34) and *r*^1 ^uniquely by (32).

## Discussions and conclusion

We have demonstrated the modeling of computational optical biopsy with the diffusion optics, the solution uniqueness properties in two typical configurations and provide explicit formulas for the reconstruction of both optical properties and source parameters. Mathematically, one single insertion will be enough to estimate the above parameters. However, physically, more measurements will guarantees the robustness of the estimates. The needle trajectory can also be dynamically restarted and optimized towards the center of the source based on the successive estimate. Further investigation on multiple point sources and multiple ball sources is undergoing. For example the double sources probelm can be handled in such a way that by using the moments defined (11) one could reduce the problem to a equivelent one with a few freedom and thus a numerical optimization technique will be used to solve the inverse problems.

## Contributions

The three authors made about equal contributions in this work.
